# Terrestrial Ecotoxicity of Plastics: Effect Factors for Life Cycle Impact Assessment

**DOI:** 10.1021/acs.est.6c03602

**Published:** 2026-07-29

**Authors:** Christina Galafton, Laura J. Zantis, Nils Thonemann, Martina G. Vijver

**Affiliations:** † Institute of Environmental Sciences, Leiden University, Einsteinweg 2, 2333 CC Leiden, The Netherlands; ‡ Business Unit Sustainability Management and Participation, Fraunhofer Institute for Environmental, Safety and Energy Technology UMSICHT, Osterfelder Str. 3, 46047 Oberhausen, Germany; § iES, Institute for Environmental Sciences, University of Kaiserslautern-Landau (RPTU), Fortstraße 7, 76829 Landau, Germany

**Keywords:** terrestrial effect factors, ecotoxicity, microplastics, soil health, life cycle assessment, impact assessment

## Abstract

Soils act as major plastic pollution sinks, with potential implications for terrestrial ecosystem services. In life cycle assessment (LCA), such impacts are quantified using characterization factors that describe the fate, exposure, and effects of emitted substances. In this study, we develop terrestrial ecotoxicity exposure-and-effect factors (XEFs) for plastics in soil for use in LCA. We compiled 680 effect concentrations (ECs) from published ecotoxicity studies involving plants and invertebrates and converted them to EC_10eq_ values. Following the USEtox framework, species-specific average EC_10eq_ values were used to construct log–normal fitted species sensitivity distribution curves, from which XEFs were derived. The resulting XEF for plastics in soil equals 0.128 [0.07; 0.30] PAF·m^3^·kg^–1^. Specific XEFs for nanoplastics ≤1 μm and plastics >1 μm were calculated at 0.921 [0.11; 13.10] and 0.126 [0.07; 0.29] PAF·m^3^·kg^–1^, respectively. Specific XEFs for natural and agricultural soil were estimated at 0.159 [0.08; 0.40] and 0.236 [0.09; 0.77] PAF·m^3^·kg^–1^, respectively. The derived XEFs are lower than aquatic and sediment XEFs by 4 and 2 orders of magnitude, respectively. Consequently, direct read-across between compartments is not justified. Overall, the proposed XEFs are compatible with the USEtox methodology and enable improved inclusion of plastic-related terrestrial ecotoxicity impacts in LCA.

## Graphical abstract

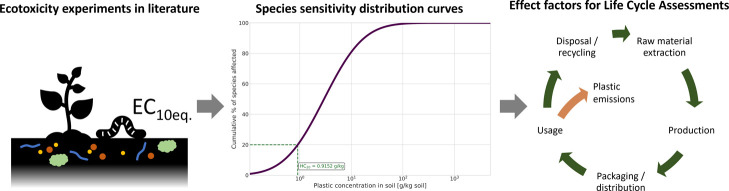

## Introduction

Soils are sinks to plastic pollution,^1,2^ receiving up to 4–23 times the amount of microplastics discharged into the ocean per year^3^ from landfills, agricultural mulching, irrigation, biosolid and compost applications, polymer-based agrochemicals, or atmospheric deposition.^4–6^ The plastic degrades slowly over the course of many years and, in some cases, decades, leading to widespread accumulation and hence pollution of soil. This pollution can affect soil function and ecosystem service provision, as well as impact the organisms that inhabit soils, including microbes, invertebrates, and plants.^5,7,8^

A common tool for evaluating environmental impacts of products and processes is life cycle assessment (LCA). In LCA, emitted substances are translated into potential impacts using so-called characterization factors. Emission-based characterization factors consist of a fate factor, an exposure factor (XF), and an effect factor (EF). The fate factor describes the redistribution, fragmentation, and degradation of the substance in the environment. The XF indicates the availability of an emitted substance to organisms and the EF quantifies the effects. The latter two can be combined into exposure-and-effect factors (XEFs) as they are sequential, multiplicative steps in the same causal chain and in practice, it can be difficult to distinguish them in experiments. Animals and plants absorb emitted substances via the outer layer of the organism. Alternatively, animals can also ingest the substances into the intestinal tract. The impacts of plastic uptake can be 2-fold: chemical (toxic) from substances leached out from the plastic emissions and/or physical particle effects.^7,9,10^ For pragmatic reasons, a joint impact by the polymer fraction and the chemicals leached from the plastic can be assumed.^11^ This assumption is consistent with life cycle impact assessment (LCIA) practice, where XEFs quantify the overall damage per unit of emission rather than isolated mechanisms. Within LCIA, XEFs represent aggregated organism-level damage rather than specific modes of toxic action. The ecotoxicological (X)­EF thus represents the chronic pressure an emitted substance exerts on an exposed ecosystem.^12,13^

XEFs have been developed for the aquatic environment, including sediments,^14–16^ as well as for the terrestrial environment.^17,18^ However, the latter are based on only 31–73 data points and 2–13 polymer types, limiting the possibilities for statistical analysis of influencing factors (e.g., the shape and size of the emission). While knowledge regarding the effects of plastic exposure on plants and soil biota is widely available,^8,9,19^ a lot of this knowledge has not yet been incorporated into XEFs.^20^ Therefore, this study aims to (i) synthesize the existing knowledge regarding terrestrial ecotoxicity effects of plastics, (ii) identify the key factors influencing the effect concentrations (ECs), and (iii) develop XEFs for terrestrial ecotoxicity effects of plastics for use in LCIA. Our contribution is 2-fold: First, we substantially expand the basis for terrestrial XEFs by collecting and analyzing a much larger data set than previously used. This enables a more robust assessment and broader comparison of plastic emissions and their impacts on soils within an LCA framework. Second, we translate this evidence into operational XEFs that support substance-level characterization and can directly be integrated into LCIA method families such as GLAM^21^ and ImpactWorld+.^22,23^ Once the method package of the Product Environmental Footprint is extended by a terrestrial ecotoxicity impact category, the XEFs can also be integrated there. Thereby, the XEFs improve both the robustness and the reliability of impact assessment and decision-making regarding plastic use restrictions, as well as the development of less harmful plastic alternatives (e.g., biodegradable formulations or reduced-additive polymers). The additional novelty of our study lies in (i) the identification of influencing factors on effect concentrations, such as emission size and soil type, and (ii) the application of different conversion factors to assess the impact of this choice on the results.

## Methodology

In LCA, toxicity impacts are usually evaluated based on the USEtox model.^12,13,24^ In USEtox, (X)­EFs are derived from species sensitivity distribution (SSD) curves constructed using measured or extrapolated chronic ECs. EC_*x*_ values indicate the concentration at which x % of a measured effect occurs or, in other words, the concentration at which the effect can be observed in x % of the sample population. In this study, XEFs are derived from the 20th percentile of an SSD curve based on EC_10_-equivalent (EC_10eq_) values in line with Owsianiak et al.^13^ EC_10eq_ values are preferred in LCIA to represent low-effect thresholds relevant for chronic ecosystem impacts. The methodology followed to obtain XEFs is visualized in Figure 1.

**Figure 1:**
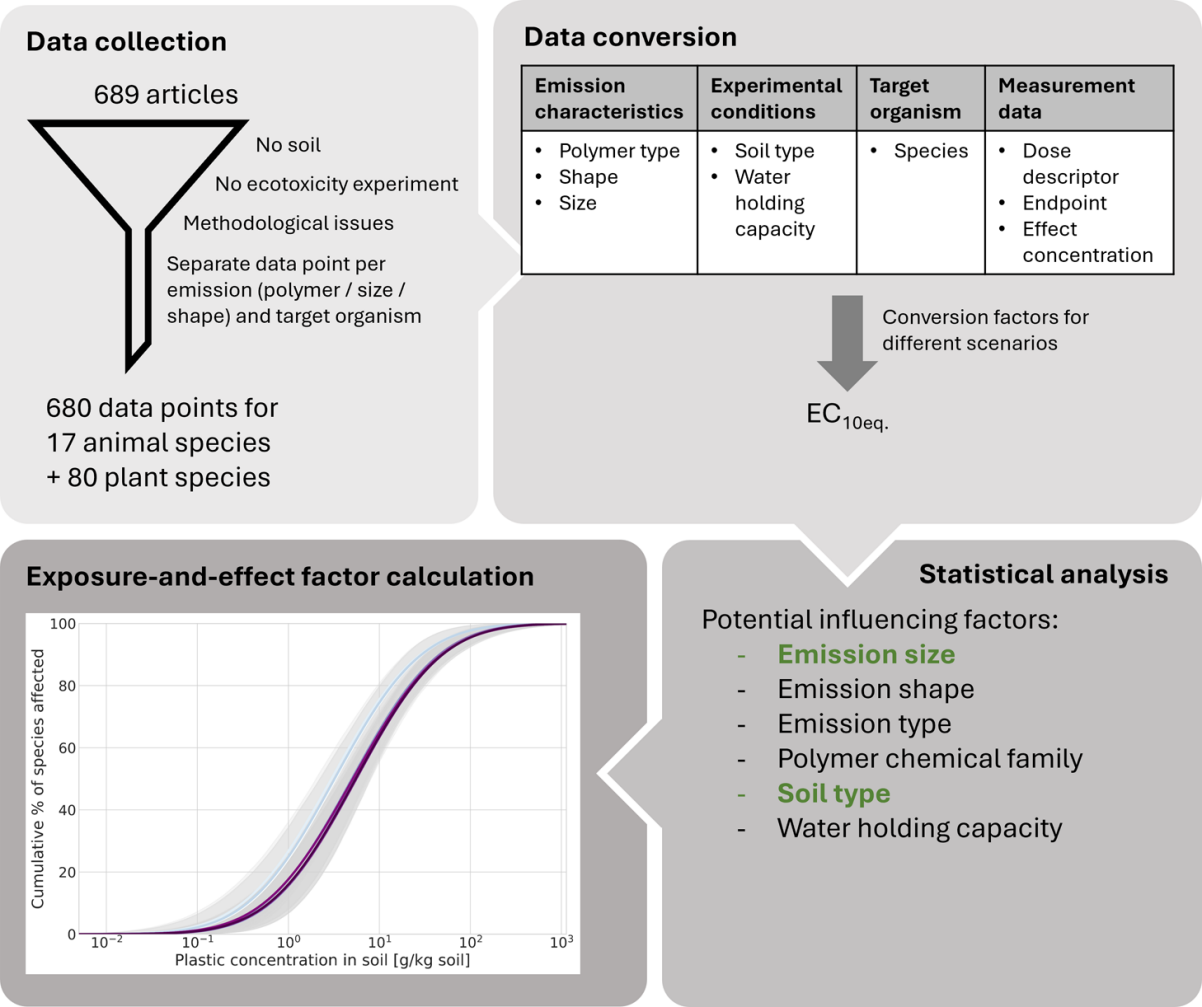
Schematic overview of the methodology used to derive terrestrial ecotoxicity exposure-and-effect factors (XEFs) for plastics. Collected ecotoxicity data are harmonized to chronic EC_10eq_ values using uncertainty factors, aggregated at the species level, and used to construct log–normal SSDs. XEFs are derived from the 20th percentile of the SSD curve (HC20_EC10eq_) and expressed as potentially affected fraction of species per unit of soil volume. XEF: exposure-and-effect factor, SSD: species sensitivity distribution, HC20_EC10eq_ = Hazard concentration that affects 20% based on ECs reported within ecotoxicity tests, EC: effect concentration.

### Data Collection

We identified relevant scientific literature by using the search term and exclusion criteria indicated in Supporting Information 1A and extracted the data presented in Supporting Information 2. In line with Aurisano et al.,^25^ we interpret LOEC values as EC_10_ values. We also recorded ECs measured for other dose descriptors and applied conversion factors, as described below.

For each data point, we recorded details regarding (i) emission characteristics: polymer type and chemical family, size, and shape, (ii) experimental conditions: soil used and water holding capacity, (iii) target organism characteristics: kingdom and species, and (iv) measurement (meta) data: dose descriptor, end point, and EC in g/kg soil. As most data concern microplastics, this group was further subdivided into a smaller and a larger size class (≤100 μm and >100 μm), following Tunali and Nowack.^17^ This stratification facilitates comparability with earlier XEF developments and reflects common size classes reported in ecotoxicological studies. The two-class approach was also chosen to ensure sufficient data density within each group, as further subdivision would have resulted in sparsely populated classes and reduced statistical robustness. While the 100 μm threshold does not represent a strict biological boundary, it provides a transparent and reproducible basis for analyzing size-dependent effects.

We distinguish natural soil collected from grasslands or forests, agricultural soil collected from agricultural fields, artificial soil that follows a standardization protocol such as the OECD guidelines (e.g.,^26–28^ ) or LUFA guidelines (LUFA is short for: Agricultural Inspection and Research Institute in Germany), artificial soil not adhering to any guidelines, and undefined. The underlying hypothesis was that the reduced biotic content of (standardized) artificial soils may influence the ECs. Likewise, background concentrations may be elevated for agricultural soils, which may also influence the results. It should be noted that the above distinctions were introduced as LCA modeling choices, rather than to imply specific toxicological thresholds or mechanisms. To allow for standardization within the SSD curve, we collected data for the same end points from all studies, focusing on conventional apical end points (lethal and sublethal). For the sublethal end points, we focused on reproduction (including growth) and neglected locomotion and fitness, as suggested by USEtox.^12^ Since every stressor can have another initial target that responds, mixing end points would impede the comparability of the data.

Apparent positive responses, such as increased growth, were classified as adverse effects. Such responses are commonly interpreted as stress-related overcompensation (hormetic stimulation), rather than genuine beneficial outcomes.^29,30^ Accordingly, any statistically significant deviation in the measured end point relative to the control condition following plastic exposure was considered an effect, irrespective of its direction. This interpretation follows a precautionary perspective consistent with ecotoxicity testing practice, where regulatory guidance generally treats any significant deviation from the control as an effect when deriving chronic ECs (e.g.,^31^), and with LCIA practice, where effect factors are based on these ECs.^24^ For studies testing several end points, we recorded each end point as a separate data entry but only used the most sensitive one per emission type and target organism, aiming to record the most sensitive end point rather than the most robust one.

Since there is no scientific consensus regarding the highest environmentally realistic concentration, we did not cut off any data points. Consequently, our data set contains data points obtained with very high concentrations, but also with very low concentrations, representing a wide range of pollution status (pristine environments up to pollution hotspots). Data collection resulted in 680 data points (one per emission type and target organism) for 17 animal species and 80 plant species. As indicated in Figure 2, most data points were collected for experiments with spherical (50%) or irregular/undefined plastic emissions (32%) and were measured for sublethal end points (86%, not shown). For plants, lethal end points were not reported at all. Most data points were collected for microplastics (36% with plastics >1 – ≤ 100 μm; 54% with plastics >100 μm – ≤ 5 mm).

**Figure 2:**
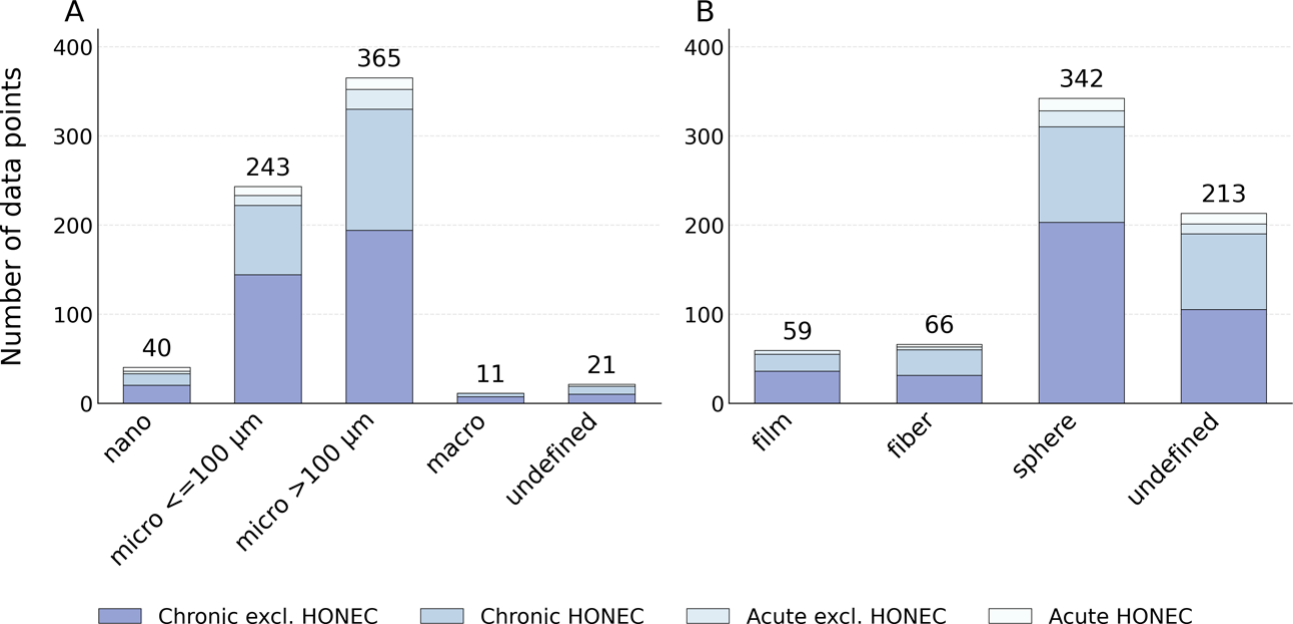
Frequency of (A) plastic emission shapes and (B) size classes used in terrestrial ecotoxicity experiments included in this study (*n* = 680). Size classes distinguish nanoplastics (≤1 μm), microplastics (>1 μm – ≤ 5 mm), and macroplastics (>5 mm), with microplastics further subdivided into ≤100 μm and >100 μm based on the largest particle dimension. Emission shapes are categorized as films, fibers, spheres, and undefined or irregular shapes.

### Data Conversion

There are several approaches to convert EC_50_ and HONEC values to EC_10eq_ values. We applied uncertainty factors based on Wigger et al.^32^ to systematically address uncertainty arising from heterogeneous ecotoxicity data. Uncertainty factors for exposure time (UF_time_) and dose descriptor (UF_dose_) were used to harmonize ECs across studies with differing experiment durations and nonequivalent effect metrics for application in an LCIA context. For EC_50_ values, we applied a UF_dose_ of 1.94 to invertebrates and 2.24 to plants based on Aurisano.^25^ This was only necessary in two cases since most studies presented EC_10_/LOEC or HONEC values. For the HONEC values, we defined three treatment options: 1. exclusion of HONEC values (‘noH’), 2. application of the same UF_dose_ as for NOEC values based on the precautionary principle and basing UF_dose_ for NOEC values on Aurisano et al.^25^ (‘H1’), and 3. application of the same UF_dose_ as for NOEC values based on the precautionary principle and assigning a conversion factor of 0.5 to NOEC values (‘H2’) in line with ECHA.^33^ All data points were classified as acute or chronic based on species-dependent thresholds.^34^ For acute values, three treatment options were defined: 1. exclusion of acute values (‘noac’), 2. application of a UF_time_ of 10 (‘UFt10’) based on Brix et al.^35^ and Wigger et al.,^32^ and 3. application of a UF_time_ of 2 (‘UFt2’) based on Rosenbaum et al.,^24^ Fantke et al.,^12^ and Lavoie et al.^14^ Each scenario represents a unique combination of a HONEC treatment and an acute-data treatment, resulting in nine scenarios in total (Table 1). Scenario names indicate the treatment of HONEC values (noH, H1, or H2) followed by the treatment of acute values (noac, UFt10, or UFt2). In the base scenario (‘noH-noac’), both HONEC and acute values were excluded.

**Table 1:** Uncertainty Factors Applied to Literature Concentrations for Conversion to Chronic EC_10eq._ Values Depending on the HONEC and Acute-Data Treatment Combination (Scenario)

scenario	UF_time_ for acute values	UF_dose_ for HONEC values
noH-noac	excluded	excluded
noH-UFt10	10	excluded
noH-UFt2	2	excluded
H1-noac	excluded	0.95 (invertebrates), 0.44 (plants)^25^
H1-UFt10	10	0.95 (invertebrates), 0.44 (plants)^25^
H1-UFt2	2	0.95 (invertebrates), 0.44 (plants)^25^
H2-noac	excluded	0.50 for all species^33^
H2-UFt10	10	0.50 for all species^33^
H2-UFt2	2	0.50 for all species^33^

We then calculated chronic EC_10eq._ from the collected data points by dividing the collected value by the product of UF_dose_ and UF_time_.

### Statistical Analysis

Data analysis and visualization were performed using Python code and OriginPro v2021b (OriginLab, USA). Statistical analyses were conducted to explore whether EC_10eq._ values varied with emission characteristics (size and shape) and material-related properties, as well as the soil used and water holding capacity. After identifying statistically significant influencing factors on EC_10eq._ values, we determined whether their combination was also statistically significant. All statistical analyses were performed for the scenario noH-noac to limit the uncertainty introduced by conversion factors. Details of the statistical tests are provided in Supporting Information 1B and 1C. The Python code is provided in Supporting Information 3.

### Exposure-and-effect Factor Calculation

We log-transformed the EC_10eq._ values and calculated means per species for each scenario as a basis for log–normal fitted SSD curves.^36^ For each curve (scenario), we calculated the arithmetic mean and standard deviation. According to Owsianiak et al.,^13^ the concentration–response slope factor, which equals the (X)­EF, lies at 20% of the SSD curve (HC20_EC10eq._). The HC20_EC10eq._ value of each scenario was, therefore, determined using eq 1 and backtransforming the results

1 $$\log\left({HC20}_{EC10eq.}\right)=\mu +\left(\sigma^{*}z_{0.2}\right)$$

where μ = the scenario-specific arithmetic mean of the mean log-transformed EC_10eq._ values per species, σ = the scenario-specific standard deviation of the mean log-transformed EC_10eq._ values per species, and z_0.2_ = −0.8416, the 20th percentile of the standard normal distribution.

To convert HC20_EC10eq._ values to XEFs expressed per unit of soil volume, a bulk soil density of 1460 kg/m^3^ was applied, following Tunali & Nowack.^17^ This value is representative of mineral soils and was adopted to ensure methodological consistency and comparability within the LCA modeling context. To consider the variation in the results, 95% confidence intervals (CIs) were calculated for the HC20_EC10eq._ values using a t-distribution.

To combine all scenarios together, we applied moment-matching (e.g.,^37^) and calculated the pooled mean μ_pooled_ and variance σ_pooled_ of the mean log-transformed EC_10eq._ values per species by applying eq 2

2 $$\mu_{pooled}=\frac{\sum {n_{i}}^{*}\mu_{i}}{\sum n_{i}}$$

where *i* = the scenario, *n*_i_ = the number of species per scenario, and μ_i_ = the scenario-specific arithmetic mean of the mean log-transformed EC_10eq._ values per species.

For the pooled scenario, following the envelope approach, the lowest lower limit of the scenarios was used as the lower 95% CI and the highest upper limit was used as the upper 95% CI. This pooled variance accounts for both within-scenario and between-scenario variability.

For each scenario, XEFs were calculated for all plastic emissions (generic), as well as all statistically significant influencing factors on the EC_10eq._ values, such as emission sizes. XEFs and CIs per scenario can be found in Supporting Information 1E.

## Results

The ecotoxicity data are strongly dominated by experiments using spherical plastic emissions (see Figure 2), likely reflecting their commercial availability and relative ease with which such particles can be produced. However, the results obtained with spherical particles do not reflect the results induced by heterogeneous shapes typically observed in plastic pollution of soils, which is dominated by fragments, films, and fibers.^38^

Most ECs were derived from experiments with microplastics larger than 1 μm and up to 5 mm in the largest dimension, whereas data for nanoplastics (≤1 μm), which in the field are predominant,^39^ remain scarce. Results showed that ECs for nanoplastics were lower than those for larger plastic particles (p = 0.0082). Given that this statement is based on a limited number of available studies and all plastic emissions fragment into smaller particles and ultimately nanoplastics, this highlights the need for additional terrestrial ecotoxicity studies focusing on nanoplastics.

### Influences on the Effect Concentrations

Since we cannot mix different ecotoxicological end point categories for the calculation of SSD curves,^40^ the determination of the SSD curves and the following analysis focused on sublethal end points only. Figure 3 indicates the distribution of the EC_10eq._ values for the base scenario (‘noH-noac’) across different classes regarding the plastic emission size, shape, chemical family, and the soil used for the ecotoxicity experiments. Most studies indicated the polymer type used for the production of the plastic used for the experiments but did not specify any additives.

**Figure 3:**
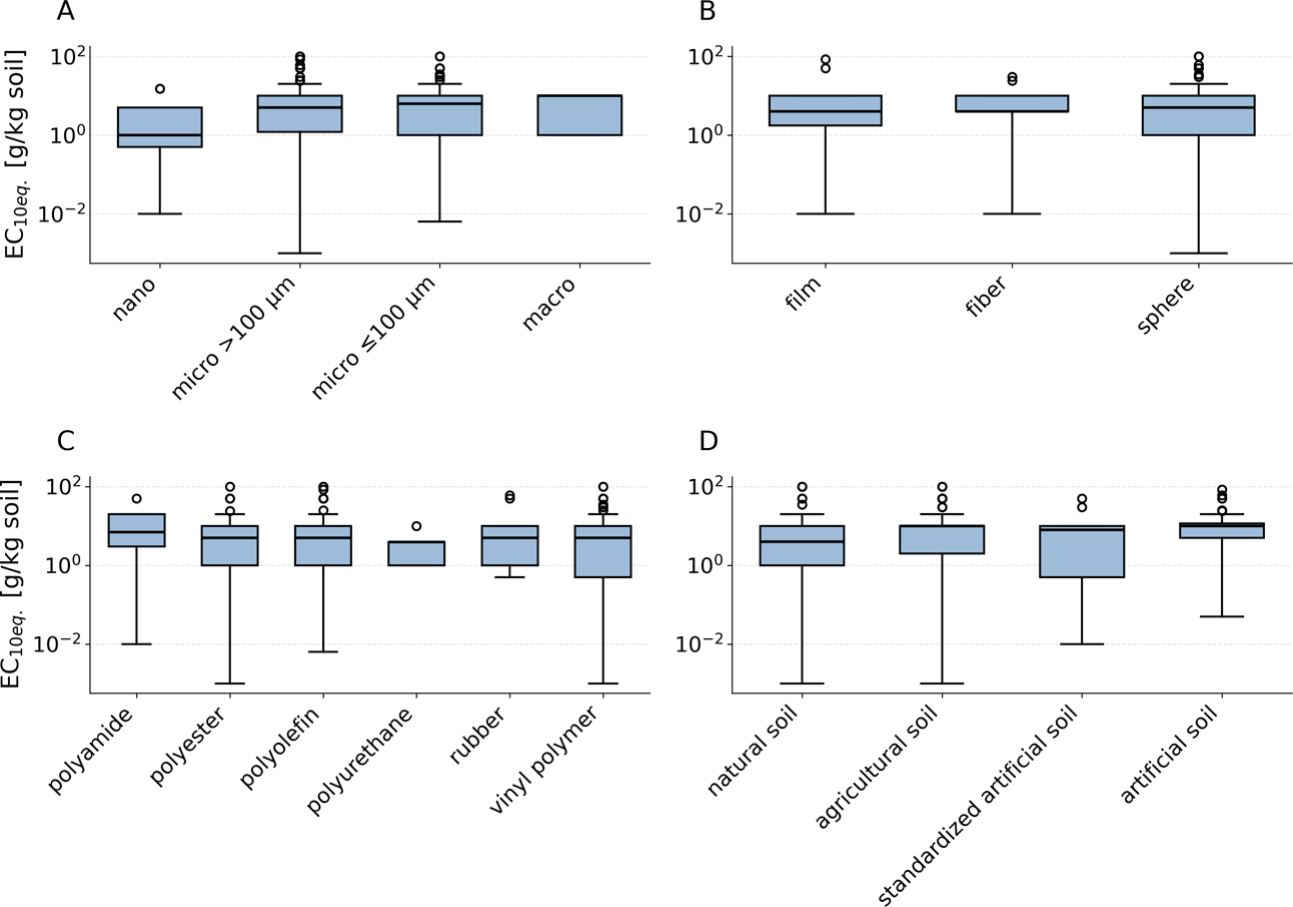
Distribution of the effect concentrations (EC_10eq._ values, expressed as g/kg soil, on a log scale) for the base scenario (‘noH-noac’) across different classes regarding (A) plastic emission sizes, (B) plastic emission shapes, (C) plastic emission chemical family, and (D) soil used in terrestrial ecotoxicity experiments included in this study. Sample sizes vary by category since irregular/undefined entries are not shown. Size classes distinguish nanoplastics (≤1 μm), microplastics (>1 μm – ≤ 5 mm) and macroplastics (>5 mm), with microplastics further subdivided into ≤100 μm and >100 μm based on the largest emission dimension. Emission shapes are categorized as films, fibers, and spheres. Boxes represent the interquartile range, the thick horizontal black line within each box indicates the median; whiskers extend to 1.5× interquartile range or the most extreme data point within that range; individual circles (○) represent outliers beyond the whisker range.

Our hypothesis testing showed that EC_10eq._ values for nanoplastics <1 μm are lower than for larger-sized plastics. While no further statistically significant effect of size class was detected using the current categorization (*p* = 0.28), the strong imbalance in group sizes, particularly for macroplastics, limits statistical power. Alternatively, mechanistically motivated size classifications or continuous representations of emission size may be more suitable to detect further size-dependent effects. For fibers, we observed a linear trend of decreasing EC_10eq._ values with increasing absolute size (Figure 4). While this may suggest that longer fibers could be more harmful, potentially due to entanglement, physical obstruction, or a greater likelihood of interacting with root or gut structures, the limited number of fiber-based studies and the dominance of spherical particles in the data set (Figure 2) must be noted. Furthermore, the trend line is statistically quite driven by the first “higher” data point, which suggests that significance here should be interpreted with caution. We found no statistically significant effect of emission shape (spheres versus films versus fibers) on EC_10eq._ values, again almost certainly constrained by unbalanced sample sizes and the scarcity of nonspherical test materials.

**Figure 4:**
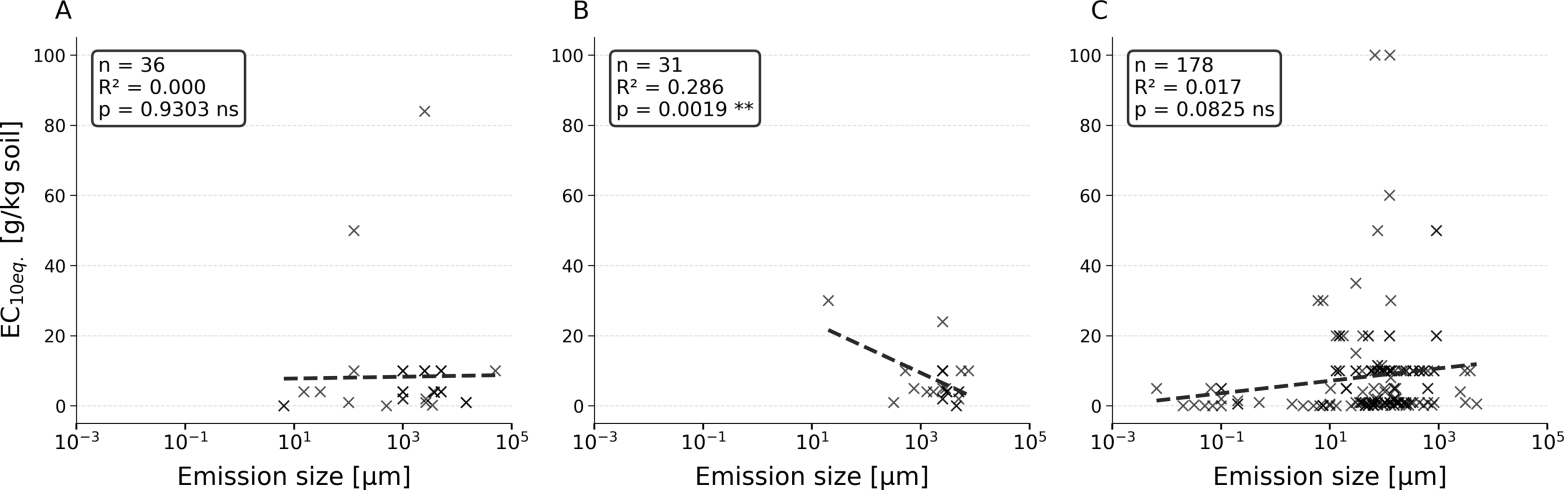
Combined influence of shape (A films, B fibers, C spheres) and size of the plastic emission (on a log-scale) on harmful effect concentrations measured as EC_10eq._ for the base scenario (‘noH-noac’) [in g/kg soil]. x marks the individual data points; the dotted line the corresponding trend line; **p < 0.01, *p < 0.05, ns not significant.

Further differences between size classes were not statistically significant, neither for individual shape categories nor for all shapes together. We, therefore, calculated a generic XEF for all emission shapes and sizes, as well as two size-specific XEFs: one for nanoplastics ≤1 μm and one for plastics >1 μm.

The Kruskal–Wallis ANOVA showed that the soil used in the experiments had a statistically significant influence on the EC_10eq._ values (p = 0.001 after Holm-Bonferroni correction). Posthoc Dunn’s test showed that the difference between natural soil and agricultural soil was statistically significant (p = 0.0084 after Holm-Bonferroni correction). Besides the generic XEF, we, therefore, calculated two specific XEFs for natural and agricultural soil. There was also a statistically significant difference between natural soil and artificial soil (p = 0.001). Although artificial soils do not fully represent natural field conditions, they provide ECs that can be interpreted as reflecting a range of soils with differing physicochemical characteristics, thereby yielding a corresponding range of ECs. Consequently, controlled laboratory experiments constitute a useful approach for evaluating the robustness and sensitivity of XEF estimates. Nevertheless, based on the lack of occurrence in nature, we did not calculate an XEF für artificial soil. Variation in experimental water holding capacity did not drive statistically significant differences in plant and invertebrate EC_10eq._ values (see Figure S2).

Based on the identified influencing factors, four combinations were defined and tested again with Kruskal–Wallis ANOVA: nanoplastics ≤1 μm in agricultural soil, plastics >1 μm in agricultural soil, nanoplastics ≤1 μm in natural soil, and plastics >1 μm in natural soil. The analysis showed that the distinction between soils was significant for plastics >1 μm but not any other distinction. Consequently, we calculated two more XEFs: for plastic emissions >1 μm in (a) agricultural soil and (b) natural soil.

### Species Sensitivity Distribution Curves

For the base scenario (noH-noac), we calculated the SSD curve displayed in Figure 5 based on 11 invertebrate species (Annelida, Arthropoda, Collembola, Nematoda) and 61 plant species (Apiaceae, Asteraceae, Brassicaceae, Cucurbitaceae, Fabaceae, Poaceae, Solanaceae, and other dicot plants and trees/woody shrubs). As indicated in brackets next to the species name on the *y*-axis, many species are represented by only one data point. The figures corresponding to the other scenarios can be found in Supporting Information 1D.

**Figure 5:**
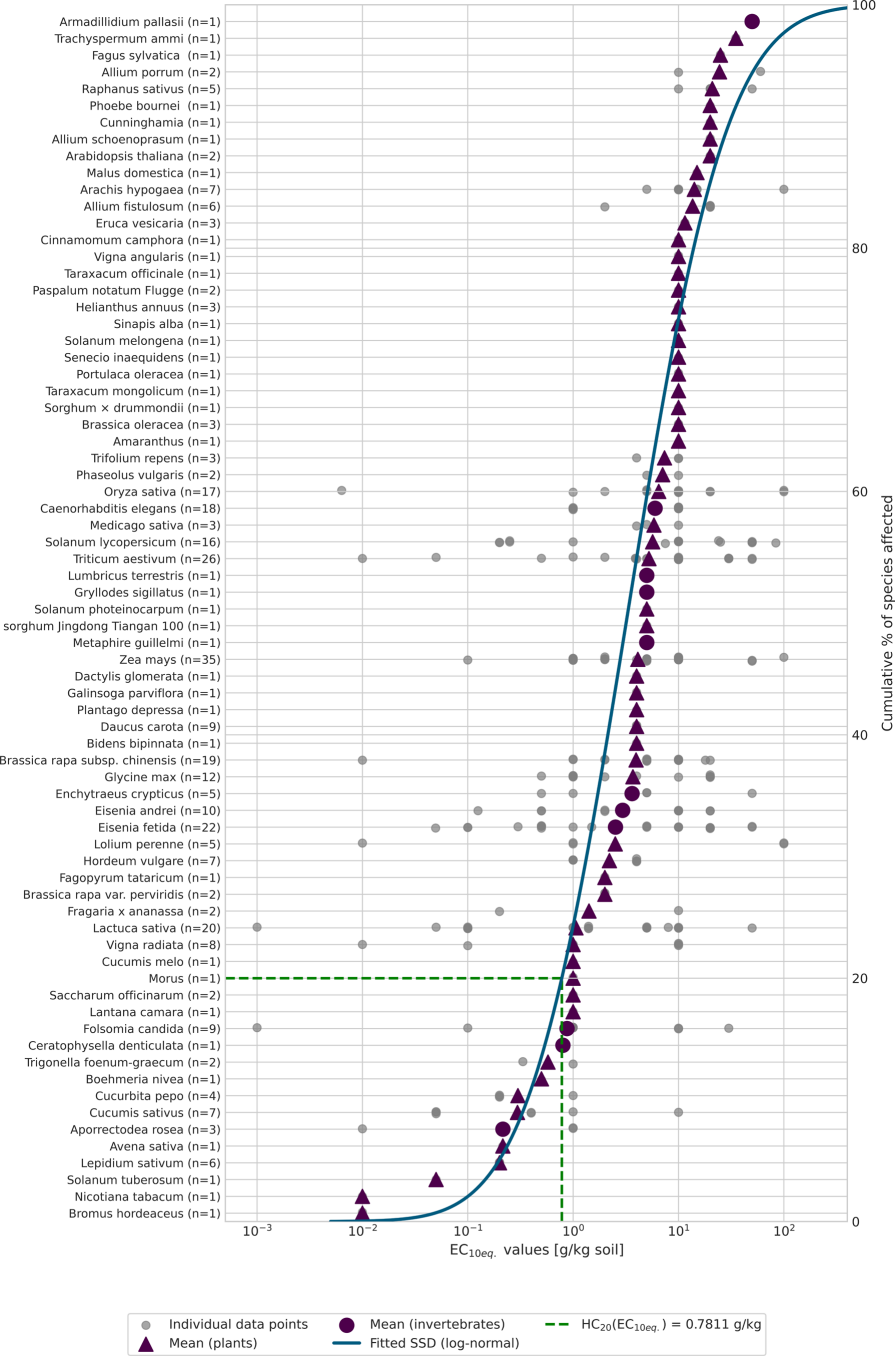
Species sensitivity distribution curve for terrestrial plastic pollution for the base scenario (“noH-noac”).

We found that the EC_10eq._ values of plants range from 0.001–100.0 g/kg soil, while EC_10eq._ values of invertebrates range from 0.001–50.0 g/kg soil. For the aquatic environment, the range of EC_10eq._ values of plants are between 0.2–1100 mg/L and for invertebrates between 0.1–1.0 mg/L when dropping ECs measured for other end points (e.g., mortality, immobilization) and using the most sensitive EC across different measurements from Corella-Puertas et al.^15^ rather than the average to be consistent to our methodology.

### Exposure-and-effect Factors

The XEFs of all scenarios together are presented in Table 2. They are based on the arithmetic means of the mean log-transformed EC_10eq._ values per species, in line with the USEtox framework. The indicated uncertainty represents an approximate log-space standard deviation derived from the 95% uncertainty envelope across scenarios, assuming lognormality. Following the results of the exploration of influencing factors, XEFs were calculated for a total of seven cases: for (i) all plastic emissions (generic), (ii) nanoplastic emissions ≤1 μm, (iii) plastic emissions >1 μm, (iv) plastic emissions in agricultural soil, and (v) plastic emissions >1 μm in agricultural soil, (vi) plastic emissions in natural soil, and (vii) plastic emissions >1 μm in natural soil. XEFs calculated for the different scenarios can be found in Supporting Information 1E. Wide confidence intervals reflect genuine ecological heterogeneity and current knowledge gaps; therefore, median effect factors should be interpreted as comparative indicators rather than precise estimates of absolute impact, and uncertainty should be explicitly considered when this impact category materially influences LCA results.

**Table 2:** Exposure-and-effect Factors for Terrestrial Ecotoxicity of Plastics Based on the Mean of the Log-Transformed EC_10eq._ Values per Species and Range of the 95% Confidence Interval (all Scenarios Pooled) [in PAF·m^3^·kg^–1^]. n.s. = not Statistically Significant

	generic (all emission sizes)	nanoplastics ≤1 μm	plastics >1 μm
plastics in soil (generic, all soils together)	0.128 [0.073; 0.300]	0.921 [0.110; 13.100]	0.126 [0.072; 0.291]
plastics in natural soil	0.159 [0.083; 0.400]	n.s	0.165 [0.085; 0.432]
plastics in agricultural soil	0.236 [0.091; 0.773]	n.s	0227 [0.083; 0.758]

## Discussion

We developed USEtox-compatible XEFs for the terrestrial ecotoxicity of plastics, based on the largest and most systematically compiled data to date, and a transparent conversion framework for experiments with different experiment durations or dose descriptors. Basing our analysis on significantly more data points than previous studies increases statistical power and improves the identification of influential parameters in regression analyses, particularly when the initial database is limited.

### Comparing Terrestrial and Aquatic XEFs of Plastics

The resulting terrestrial XEFs are much lower than those for the aquatic environment (22.6 PAF·m^3^·kg^– 1^;^41^ 1067.5 PAF·m^3^·kg^–1^ ^15^) and sediment (16.2 PAF·m^3^·kg^–1^ ^16^), but in the same order of magnitude as the previously developed XEFs for the terrestrial environment (0.616 PAF·m^3^·kg^–1^ ^17^). However, the aforementioned studies used data obtained for a combination of different ecotoxicological end points (mortality, reproduction, growth, feeding, immobilization), which limits the comparability to our study. Besides, the aforementioned studies address only the physical effects on biota based on experiments with virgin polymers. They aim to exclude the ecotoxicological effects of leachates but acknowledge that virgin polymers could contribute to ecotoxic effects via residual, nonpolymerized monomers. The XEFs presented here address both physical and ecotoxic effects. Similarly, a comparison to the terrestrial XEFs developed by Vázquez-Vázquez et al.^18^ is not possible due to differences in the methodology. Concluding, a read-across between the different environmental compartments for missing data is not straightforward. This also puts into question whether conversion factors for different dose descriptors and acute values developed for the aquatic environment apply to the terrestrial environment.

### Influential Parameters Affecting the XEFs of Plastics

As emphasized before, Vázquez-Vázquez et al.^18^ and Tunali & Nowack^17^ derived XEFs for the terrestrial environment based on small data sets. Their reported distinctions related to polymer type could not be confirmed within our analysis based on our extended data set. Explicitly, our results did not prove significant differences between plastics and rubber (tire wear) nor among chemical polymer families in terms of the EC_10eq_ values. The lacking influence of the chemical family of the polymer on ECs suggests that toxicity differences arising from additive formulations within a given polymer type may be larger than differences attributable to the base polymer itself.^42^ This is in line with findings regarding the effects of plastics on aquatic organisms.^14–16^

We did, however, find a statistically significant influence of the emission size on the EC_10eq_ values. This leads to an XEF for nanoplastics that is 7 times the XEF for plastics >1 μm in soil, which is, however, still in the same order of magnitude. While the influence of the soil used in the experiments was also statistically significant for the EC_10eq_ values (natural versus agricultural soil: p = 0.0084), this likely indicates that exposure rather than effects is different in agricultural and natural soil, although both aspects are combined in our XEFs. Agricultural soils typically contain higher organic matter content, different microbial communities adapted to anthropogenic inputs, and potentially elevated background concentrations of microplastics.^6^ Combined, these factors may influence plastic bioavailability, degradation rates, and additive leaching. Nevertheless, the XEFs for agricultural soil and natural soil are very similar, and the 95% CIs largely overlap.

Likewise, the difference between natural and artificial soils (*p* = 0.001) was expected, as standardized artificial soils intentionally minimize biotic complexity to ensure reproducibility.^26–28^ However, this puts the ecological relevance of laboratory studies in artificial media for the derivation of ecotoxicological XEFs into question. The main benefit may, instead, lie in providing valuable mechanistic insights and standardized comparisons, but extrapolation to field conditions is not straightforward. Including experiments with artificial soil in the XEF calculation reduces the XEF compared to natural and agricultural soil (see Table 2), possibly leading to an underestimation of the XEF under natural conditions. Nevertheless, the XEFs are all in the same order of magnitude. Although no statistically significant differences were detected, the method used to create the plastic exposure in the experiments (e.g., using commercially available particles versus shredded or milled macroplastic emissions) may influence the observed ranges.^100^ Overall, the soil-specific XEFs indicate that exposure is different in different soils, which should be further investigated in the future.

These deviations from previous XEF findings prove that expanding the underlying data set provides a more robust and comprehensive foundation for analysis. It is known that increasing the data set size enhances statistical power and improves the identification of influential parameters within regression analyses.

### Methodological Implications and Uncertainty

Out of the 680 extracted data points, 630 (93%) were obtained with virgin materials that were cut, ground, or milled to resemble plastic emissions. Nevertheless, these materials fail to mimic longer-term processes such as additive leaching or population with eco-corona. More research is needed with aged materials (both artificial aging and previous exposure to nature) to validate the previous findings. Besides, future ecotoxicity experiments should specify the additives used for the plastic emission to enable further insight into the causes of the impacts (physical or chemical).

While the approach using uncertainty factors (UF_time_ and UF_dose_) followed established practice for engineered nanomaterials,^32,43^ the appropriateness of these factors for (terrestrial) plastics requires validation. The scenario analysis regarding the conversion factors demonstrates the sensitivity of XEF calculations to these assumptions (Supporting Information 1F). Across the nine scenarios, XEFs showed moderate but systematic variability, reflecting uncertainty associated with data selection and conversion assumptions rather than random noise. The 95% CIs overlapped substantially across scenarios, suggesting that alternative treatments of acute and HONEC data influence absolute XEF values but do not alter their order of magnitude or relative interpretation. This robustness supports the use of a single generic XEF (0.128 [0.073; 0.300] PAF·m^3^·kg^–1^).

Especially for smaller emissions, the number of particles used in the experiments or their surface area might be more accurate parameters to assess toxicity than mass-based concentrations. Nevertheless, these parameters are barely reported in ecotoxicity experiments. Besides, the USEtox methodology results in characterization factors per emitted mass. Consequently, the methodology would need adaptation to account for surface-related interactions.

Since PAF-based XEFs indicate the potential fraction of affected species compared to other substances, they do not directly quantify damage on an end point level, such as ecosystem function loss or the number of species to go extinct. XEFs as applied in LCIA are derived from a limited number of species and predominantly from single-species experiments conducted under constant, controlled exposure conditions that do not fully capture natural complexity. Consequently, the PAF-based XEFs are a common way to quantify potential environmental impacts in LCIA but need further explanation and interpretation when used in other contexts, such as risk assessment.

### XEFs to be Used for LCIA Practice

The generic XEF of 0.128 [0.073; 0.300] PAF·m^3^·kg^–1^ is appropriate for the majority of LCA studies, including screening assessments as well as detailed studies where plastic emissions are not the primary focus of the investigated product system. This generic XEF considers different approaches for data selection and conversion for shorter experiments and experiments that measured different dose descriptors. The confidence interval indicated for this XEF represents the widest interval of all investigated scenarios combined, avoiding underrepresenting the level of uncertainty. At the same time, the moderate width of the confidence interval (1 order of magnitude) is unlikely to affect the relative contribution of the emission’s terrestrial ecotoxicity to the overall impacts in cases where this impact category is not dominant. Size- and/or soil-specific XEFs (see Table 2) are preferable when the study explicitly focuses on plastic-emitting product systems (e.g., comparing agricultural mulching film alternatives or biodegradable versus conventional polymers), the emission size and receiving environment are known, and inventory data is correspondingly detailed. Although the XEFs for the different soils are in the same order of magnitude, using the soil-specific XEFs may make a difference in comparative analyses depending on the case study, especially when plastic emissions are dominated by one soil context (e.g., agricultural mulching films versus forest road runoff or different pH levels or organic matter content). Currently, corresponding fate factors for different soil types are lacking. Given that the confidence intervals of the XEFs overlap substantially across soil types and size classes above 1 μm (Table 2), results should be interpreted in terms of order-of-magnitude differences rather than absolute values. If the ranking of alternatives changes when applying lower versus upper confidence limits, the results should be interpreted as indicative rather than decisive for decision-making. In such cases, sensitivity or uncertainty analyses are recommended to assess the robustness of comparative conclusions.

The fact that ultimately all plastic fragments into nanoplastics implies that over the entire lifetime of a plastic emission in the environment, the XEF applied may need to change (using the XEF of 0.126 PAF·m^3^·kg^–1^ for larger emissions and the XEF of 0.921 PAF·m^3^·kg^–1^ once they have fragmented into pieces with all dimensions ≤1 μm). This could be considered when combining the developed XEFs with fate factors to characterization factors, as nanoplastics are increasingly recognized as the most bioavailable and potentially hazardous fraction,^39^ and plastic emissions are ultimately expected to fragment into nanoplastics over time. Nevertheless, introducing this concept to the characterization factors would require increased representation of nanoplastics in the data set first, to allow for a more robust hypothesis testing regarding the influence of emission size.

Although the interaction between emission size and soil type was also statistically significant in some cases (see above), the corresponding XEFs are derived from considerably fewer species and less robust data sets. Therefore, further investigation is recommended before these XEFs are applied more generally in LCAs. Overall, this work provides a transparent and USEtox-consistent foundation for incorporating terrestrial plastic ecotoxicity into LCIA, while highlighting priority data gaps, particularly for soil-specific processes, which must be addressed to further refine XEF development.

## Supplementary Material



## Data Availability

Raw and converted data collected during the literature review that were used for our analysis, as well as the Python code used for analysis and visualization, can be found in the following repository: Galafton, C., Zantis, L. J., Thonemann, N., & Vijver, M. G. (2026). Terrestrial ecotoxicity of plastics: Effect factors for life cycle impact assessment—Data retrieved from ecotoxicity experiments and Python code for analyses and effect factor calculation (v1.0) (https://doi.org/10.5281/zenodo.18743274).

## Notes

Additional details regarding the methodology (literature review and statistical analysis) and results (SSD curves and effect factors for each scenario), including figures and tables (PDF)

## Abbreviations

CI: confidence interval
EC: effect concentration
EF: effect factor
LCA: life cycle assessment
LCIA: life cycle impact assessment
PAF: potentially affected fraction of species
SSD: species sensitivity distribution
XEF: exposure-and-effect factor
