# Supplementary material for: Terrestrial Ecotoxicity of Plastics: Effect Factors for Life Cycle Impact Assessment
Source: Environ Sci Technol. 2026 Jul 29;60(31):21522–31. doi: 10.1021/acs.est.6c03602 (PMC13472140; doi:10.1021/acs.est.6c03602)
Supplement: Supplementary file 1 [file es6c03602_si_001.pdf]

Supporting Information 1

Title: Terrestrial Ecotoxicity of Plastics: Effect Factors for Life Cycle Impact Assessment

Authors: Christina Galafton[*, 1, 2], Laura J. Zantis[1, 3], Nils Thonemann[1], Martina G. Vijver[1]

[1] Institute of Environmental Sciences, Leiden University, Einsteinweg 2, 2333 CC, Leiden, The Netherlands

[2] Business Unit Sustainability Management and Participation, Fraunhofer Institute for Environmental, Safety and Energy Technology UMSICHT, Osterfelder Str. 3, 46047, Oberhausen, Germany

[3] iES, Institute for Environmental Sciences, University of Kaiserslautern-Landau (RPTU), Fortstraße 7, 76829 Landau, Germany

[*]corresponding author: c.galafton@cml.leidenuniv.nl

Content

SI1A: Literature search..2

SI1B: Methods for statistical analysis 4

SI1C: Results of the statistical analyses 5

SI1D: Species sensitivity distribution curves 6

SI1E: Exposure-and-effect factors 8

References..10

12 pages

3 figures

4 tables

SI1A: Literature search

We obtained the effect concentrations by searching scientific literature in the databases Web of Science and Scopus in February 2025 by using the following search term in the article titles, abstracts, and keywords:

> *(microplastic* OR nanoplastic* OR macroplastic*)*
>
> *AND (ecotoxic* OR toxic* OR effect* OR impact* OR response)*
>
> *AND (soil OR terrestrial)*
>
> *AND (mortality OR survival OR reproduction OR development OR growth OR germination OR length OR biomass OR leaves)*

To reduce the number of irrelevant hits, we excluded articles with the words 'waste', 'wastewater', or 'human' in the article title, abstract, or keywords. Besides, we focused on peer-reviewed research articles and excluded articles not written in English. During the screening of the titles and abstracts, we excluded duplicates from the two databases, studies that did not describe an empirical experiment, and experiments conducted with species other than terrestrial or without soil (e.g., hydroponics). Hydroponic studies were excluded because effect concentrations cannot be expressed per soil mass. In the next step, we extracted data from the remaining articles (see SM xx). Since we interpret LOEC values (the lowest tested concentration with effects that differ statistically significantly from the control) as EC_{10} values (2019), we prioritized extracting EC_{10} and LOEC values, in order to avoid extrapolation and reduce the uncertainty of the results. If EC_{10} and LOEC values were not presented, we extracted EC_{50} values or, if no effects were found or effects found were not statistically significant ($p>0.05$), recorded the highest concentration tested as HONEC.

The search rendered 528 articles in Web of Science and 511 in Scopus, including 354 duplicates. After reading the titles, abstracts and, in many cases, full texts of the remaining 685 articles, 459 were excluded for the following reasons: they referred to the aquatic ecosystem (18%), did not describe an experiment (37%), conducted an experiment without soil (10%), did not measure apical endpoints (5%), or contained other methodological decisions that were incompatible with our research (29%). The exclusion process is displayed in Figure S1.

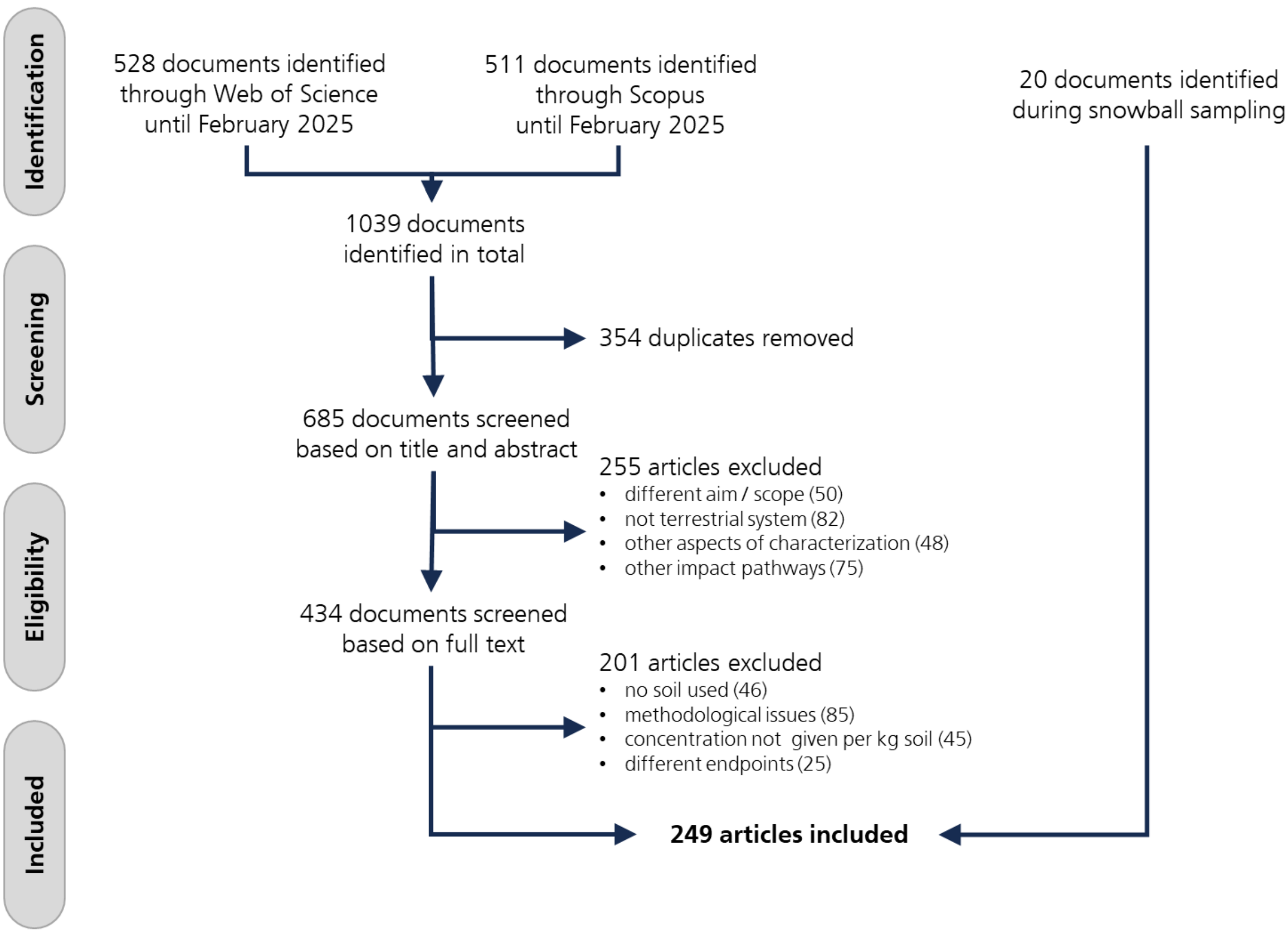

Figure S1: Systematic literature research and exclusion process

SI1B: Methods for statistical analysis

After dropping the datapoints regarding lethal endpoints, as well as acute and HONEC values, all recorded data were tested using the Shapiro-Wilk test to check for normality across all species and per species, where neither was the case except for Medicago sativa (n=3). We then applied the Brown-Forsythe test (median-centered, for data that is not normally distributed) and found out that variances between the species are not significantly different (p=0.78 for the base case). For further statistical analysis, we excluded data sets with undefined values for the respective analyzed factor (polymer type, size class, shape, or soil type) to ensure clear categorical comparisons per factor. In all analyses, $p < 0.05$ was considered significantly different. We conducted three types of statistical comparisons across all species:

(i) hypothesis-driven pairwise comparisons between spherical vs. non-spherical emissions; nanoplastics <1 µm vs. >1 µm, and macroplastics >5mm vs. <5 mm), using Mann-Whitney U tests without correction for multiple testing to avoid inflating Type II error;

(ii) exploratory omnibus tests to examine whether $EC_{10eq.}$ values differed depending on the polymer source (plastics, rubber), chemical family (polyolefins, polyesters, polyurethane, vinyl polymers, polyamide, rubber), size (nanoplastics <1 µm, microplastics <100 µm, microplastics >100 µm, macroplastics >5 mm), shape (film, fibers, spheres), and soil type (natural, agricultural, artificial), by using Kruskal-Wallis ANOVA with Holm-Bonferroni correction (Holm 1979) to control the error rate, and

(iii) post-hoc pairwise comparisons for factors showing significant Kruskal-Wallis results ($p < 0.05$) to identify which specific groups differed using Dunn's test with Bonferroni correction.

SI1C: Results of the statistical analyses

Table S1 shows the results of the Kruskal-Wallis ANOVA testing with Holm-Bonferroni correction for the factors type, chemical family, size, and shape of the emission, as well as the soil type used in the experiments.

Table S1: Results of the Kruskal-Wallis ANOVA with Holm-Bonferroni correction per factor

Factor	No of groups	Groups	Total n	Group sizes	H_statistic	p-value (Holm adjusted)	Significant?
Type	2	plastic, rubber	351	340, 11	0.1133	1	no
Chemical family	6	Vinyl polymer, Polyolefin, Polyamide, Polyester, Rubber, Polyurethane	335	77, 153, 8, 81, 11, 5	2.5717	1	no
Size	4	nano, micro ≤100 µm, micro >100 µm, macro	341	17, 138, 179, 7	7.0589	0.3502	no
Shape	3	spherical, Fiber, Film	249	182, 31, 36	0.8925	1	no
Soil type	4	natural soil, standardized artificial soil, agricultural soil, artificial soil	314	148, 21, 104, 41	19.5693	0.0013	YES
Soil type x size	4	nano_agricultural, nano_natural, non-nano_agricultural, non-nano_natural	246	5, 7, 95, 139	18.9136	0.0003	YES

There was no statistically significant influence of the water holding capacity used in the experiments on the effect concentrations for plants and invertebrates (see Figure S2).

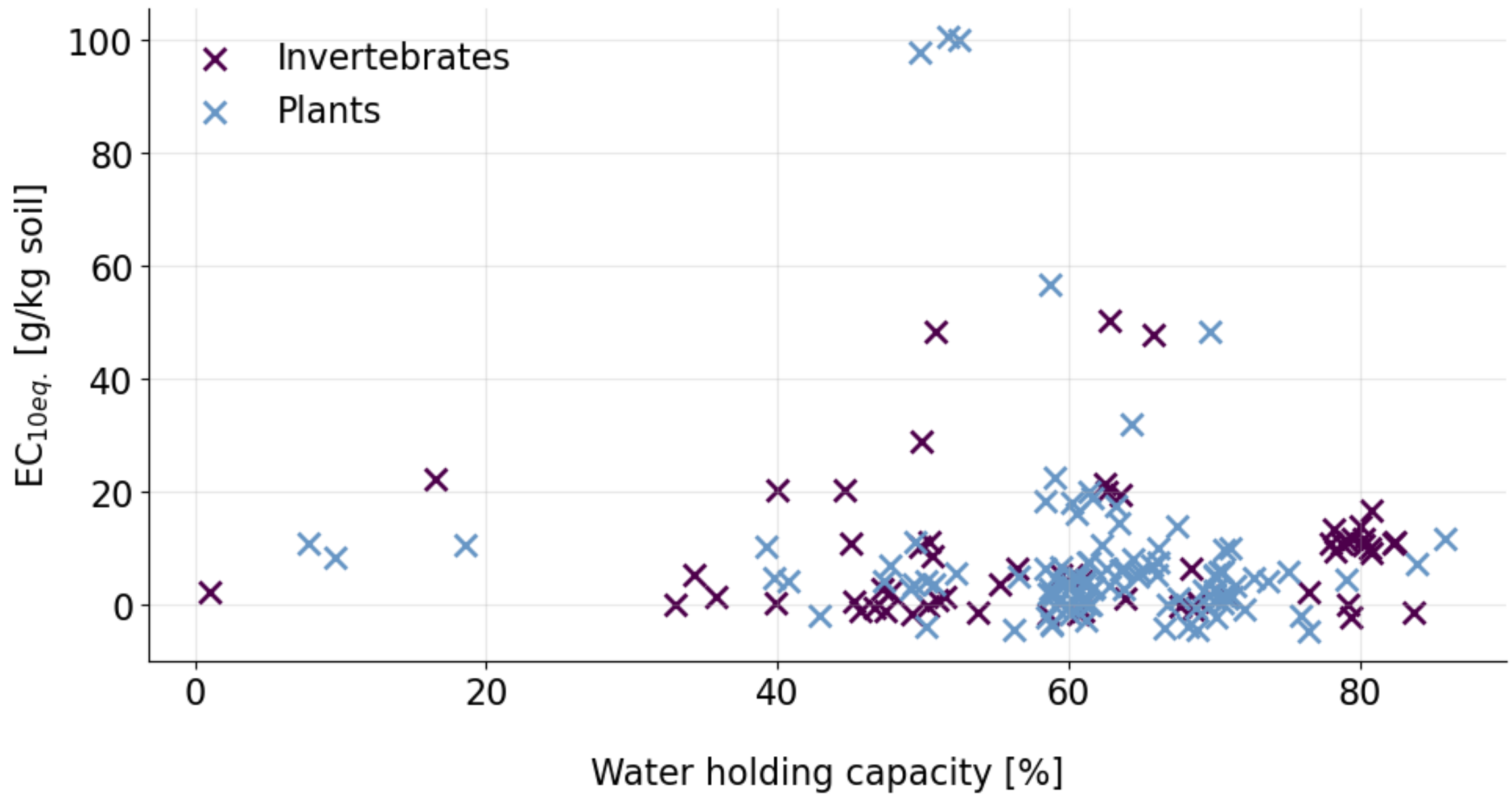

Figure S2: Correlation between water holding capacity [%] in the experiments (x-axis) and the measured harmful effect concentration [g/kg soil] (y-axis) for the base scenario ('noH-noac')

SI1D: Species sensitivity distribution curves

Figure S3 shows the SSD curves of all scenarios except for the base scenario (noH-noac), which is included in the main text.

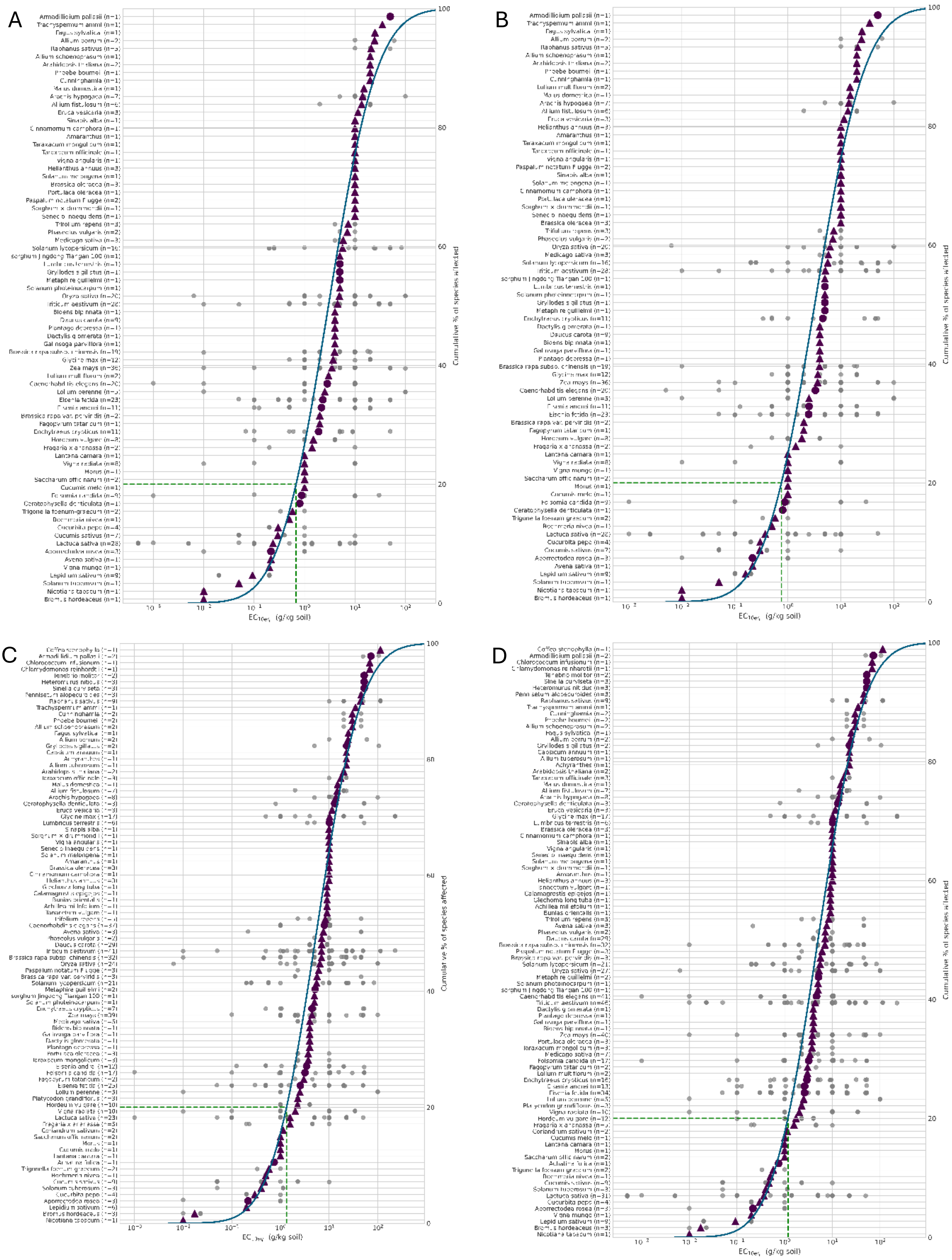

(Figure S3 continues on page 7)

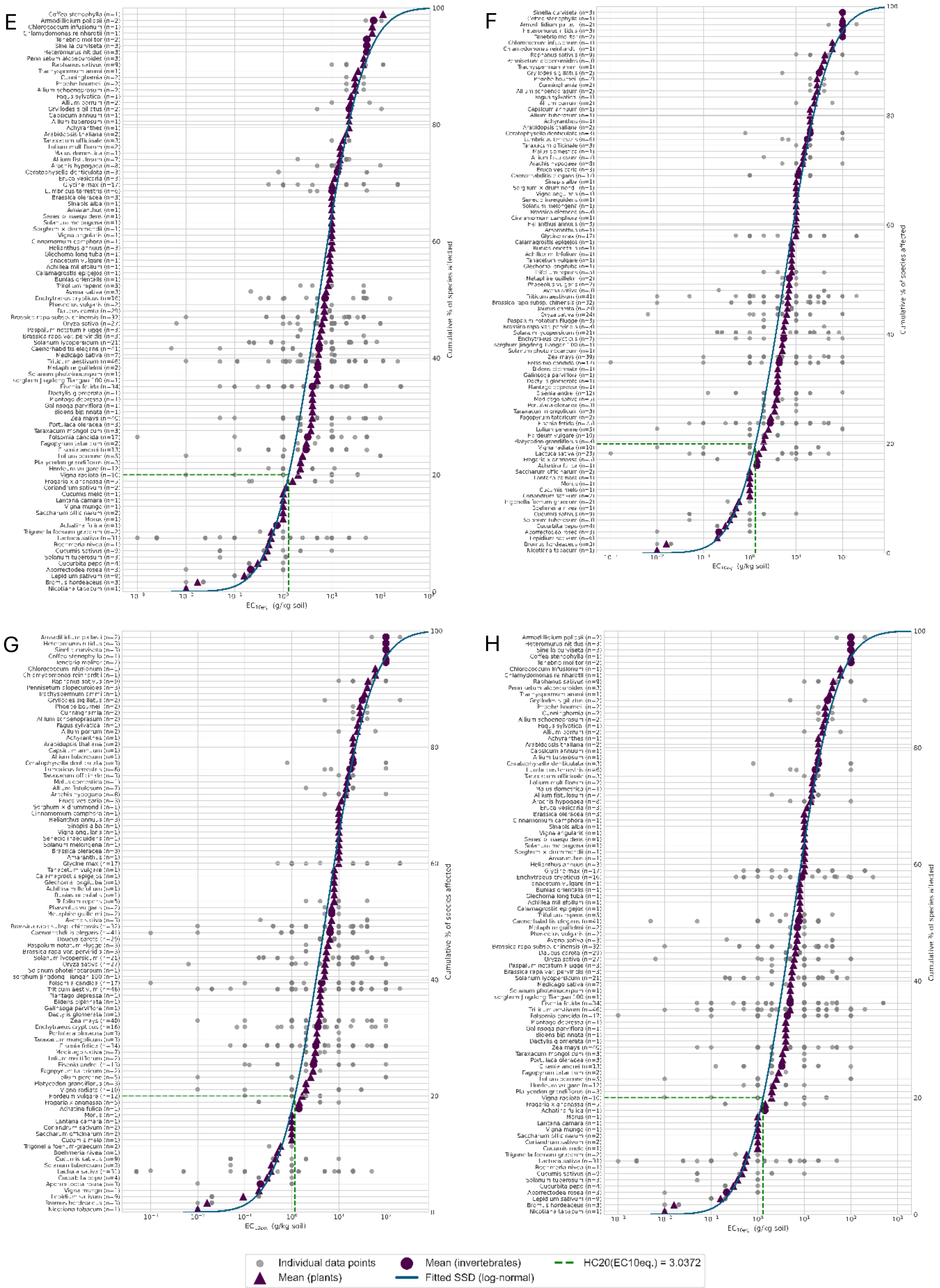

Figure S3: Species sensitivity distribution curves of the scenarios (A: noH-UFt10, B: noH-UFt2, C: HN-noac, D: HN-UFt10, E: HN-UFt2, F: H2L-noac, G: H2L-UFt10, H: H2L-UFt2)

SI1E: Exposure-and-effect factors

Table S2 displays the generic XEFs calculated for the different scenarios including the geometric mean and 95% confidence interval.

Table S2: Exposure-and-effect factors indicating the terrestrial ecotoxicity of plastics in soil for the different scenarios (generic XEF independent of emission size and soil used)

Scenario	No. species	μ log10($EC_{10eq.}$) *[g/kg soil]*	σ log10($EC_{10eq.}$) *[g/kg soil]*	XEF *[PAF·m³·kg⁻¹]*	Lower 95% CI *[PAF·m³·kg⁻¹]*	Upper 95% CI *[PAF·m³·kg⁻¹]*
**Pooled**	**96**	**0.662**	**0.753**	**0.128**	**0.073**	**0.300**
noH-noac	76	0.518	0.743	0.175	0.119	0.259
noH-UFt10	78	0.474	0.763	0.202	0.136	0.300
noH-UFt2	78	0.508	0.747	0.181	0.123	0.267
H1-noac	94	0.738	0.727	0.103	0.073	0.145
H1-UFt10	96	0.699	0.751	0.117	0.083	0.167
H1-UFt2	96	0.730	0.731	0.105	0.075	0.148
H2-noac	94	0.748	0.741	0.103	0.073	0.146
H2-UFt10	96	0.709	0.763	0.117	0.082	0.168
H2-UFt2	96	0.740	0.745	0.105	0.075	0.149

CI: confidence interval

Since there is no scientific consensus regarding the highest environmentally realistic concentration, we did not cut off any datapoints. Nevertheless, to indicate the impact this choice has on the results, Table S3 presents the XEFs when excluding datapoints obtained with concentrations higher than 10g plastics / kg soil. The results change by less than an order of magnitude.

Table S3: Exposure-and-effect factors indicating the terrestrial ecotoxicity of plastics in soil for the different scenarios (generic XEF independent of emission size and soil used), when excluding datapoints obtained with concentrations higher than 10g plastics / kg soil

Scenario	No. species	μ log10($EC_{10eq.}$) *[g/kg soil]*	σ log10($EC_{10eq.}$) *[g/kg soil]*	XEF *[PAF·m³·kg⁻¹]*	Lower 95% CI *[PAF·m³·kg⁻¹]*	Upper 95% CI *[PAF·m³·kg⁻¹]*
**Pooled**	**79**	**0.375**	**0.688**	**0.219**	**0.131**	**0.451**
noH-noac	67	0.324	0.708	0.256	0.172	0.381
noH-UFt10	68	0.275	0.731	0.300	0.200	0.451
noH-UFt2	68	0.303	0.710	0.270	0.182	0.401
H1-noac	78	0.428	0.664	0.185	0.131	0.262
H1-UFt10	79	0.384	0.688	0.215	0.151	0.306
H1-UFt2	79	0.412	0.668	0.194	0.137	0.273
H2-noac	78	0.427	0.661	0.185	0.131	0.260
H2-UFt10	79	0.383	0.684	0.214	0.150	0.304
H2-UFt2	79	0.411	0.664	0.193	0.137	0.271

Table S4 displays the XEFs calculated for the different scenarios for plastics in natural and agricultural soil including the mean and 95% confidence interval.

Table S4: Exposure-and-effect factors indicating the terrestrial ecotoxicity of plastics in natural versus agricultural soil for the different scenarios (n.s. = not statistically significant)

Scenario	Emission size	Plastics in soil (generic; independent of soil used)	Plastics in agricultural soil	Plastics in natural soil
**All scenarios pooled**	**Independent of emission size**	**0.128 [0.073; 0.3]**	**0.236 [0.091; 0.773]**	**0.159 [0.083; 0.4]**
	**Nanoplastics ≤1µm**	**0.921 [0.11; 13.1]**	**n.s.**	**n.s.**
	**Plastics >1µm**	**0.126 [0.072; 0.291]**	**0.227 [0.087; 0.758]**	**0.165 [0.085; 0.432]**
noH-noac	Independent of emission size	0.175 [0.119; 0.259]	0.268 [0.119; 0.600]	0.226 [0.136; 0.373]
	Nanoplastics ≤1µm	0.903 [0.209; 3.91]	n.s.	n.s.
	Plastics >1µm	0.170 [0.114; 0.253]	0.242 [0.107; 0.551]	0.238 [0.141; 0.401]
noH-UFt10	Independent of emission size	0.202 [0.136; 0.3]	0.339 [0.149; 0.773]	0.240 [0.144; 0.4]
	Nanoplastics ≤1µm	2.650 [0.533; 13.1]	n.s.	n.s.
	Plastics >1µm	0.194 [0.13; 0.291]	0.324 [0.138; 0.758]	0.255 [0.151; 0.432]
noH-UFt2	Independent of emission size	0.181 [0.123; 0.267]	0.307 [0.137; 0.690]	0.228 [0.138; 0.378]
	Nanoplastics ≤1µm	1.520 [0.38; 6.1]	n.s.	n.s.
	Plastics >1µm	0.174 [0.117; 0.259]	0.287 [0.125; 0.661]	0.241 [0.143; 0.407]
H1-noac	Independent of emission size	0.103 [0.073; 0.145]	0.194 [0.094; 0.400]	0.129 [0.083; 0.198]
	Nanoplastics ≤1µm	0.467 [0.12; 1.82]	n.s.	n.s.
	Plastics >1µm	0.101 [0.072; 0.143]	0.184 [0.089; 0.378]	0.133 [0.085; 0.207]
H1-UFt10	Independent of emission size	0.117 [0.083; 0.167]	0.233 [0.112; 0.486]	0.138 [0.089; 0.214]
	Nanoplastics ≤1µm	1.08 [0.227; 5.08]	n.s.	n.s.
	Plastics >1µm	0.116 [0.082; 0.166]	0.231 [0.110; 0.485]	0.142 [0.090; 0.224]
H1-UFt2	Independent of emission size	0.105 [0.075; 0.148]	0.214 [0.103; 0.441]	0.132 [0.085; 0.204]
	Nanoplastics ≤1µm	0.74 [0.176; 3.12]	n.s.	n.s.
	Plastics >1µm	0.104 [0.074; 0.147]	0.208 [0.101; 0.430]	0.136 [0.087; 0.213]
H2-noac	Independent of emission size	0.103 [0.073; 0.146]	0.189 [0.091; 0.393]	0.130 [0.084; 0.200]
	Nanoplastics ≤1µm	0.415 [0.11; 1.56]	n.s.	n.s.
	Plastics >1µm	0.102 [0.072; 0.145]	0.181 [0.087; 0.377]	0.134 [0.086; 0.209]
H2-UFt10	Independent of emission size	0.117 [0.082; 0.168]	0.227 [0.108; 0.478]	0.139 [0.090; 0.216]
	Nanoplastics ≤1µm	0.998 [0.215; 4.62]	n.s.	n.s.
	Plastics >1µm	0.117 [0.082; 0.168]	0.227 [0.107; 0.482]	0.143 [0.091; 0.226]
H2-UFt2	Independent of emission size	0.105 [0.075; 0.149]	0.209 [0.101; 0.434]	0.133 [0.086; 0.205]
	Nanoplastics ≤1µm	0.690 [0.166; 2.86]	n.s.	n.s.
	Plastics >1µm	0.104 [0.074; 0.148]	0.205 [0.098; 0.429]	0.137 [0.088; 0.214]

References

Agez, Maxime; Muller, Elliot; Bulle, Cécile; Debarre, Laura; Seitfudem, Georg; Viveros Santos, Ivan; Duval, Lisa (2024): IMPACT World+ / a globally regionalized method for life cycle impact assessment: Zenodo.

Aurisano, Nicolò; Albizzati, Paola Federica; Hauschild, Michael; Fantke, Peter (2019): Extrapolation Factors for Characterizing Freshwater Ecotoxicity Effects. In *Environmental Toxicology and Chemistry* 38 (11), pp. 2568–2582. DOI: 10.1002/etc.4564.

Bläsing, Melanie; Amelung, Wulf (2018): Plastics in soil: Analytical methods and possible sources. In *Sci Total Environ* 612, pp. 422–435. DOI: 10.1016/j.scitotenv.2017.08.086.

Borenstein, Michael; Hedges, Larry V.; Higgins, Julian P. T.; Rothstein, Hannah R. (Eds.) (2009): Introduction to Meta-Analysis. With assistance of Michael Borenstein, Larry V. Hedges, Julian P. T. Higgins, Hannah R. Rothstein.

Brix, K. V.; DeForest, D. K.; Adams, W. J. (2001): Assessing acute and chronic copper risks to freshwater aquatic life using species sensitivity distributions for different taxonomic groups. In *Environmental Toxicology and Chemistry* 20 (8), pp. 1846–1856.

Bulle, Cécile; Margni, Manuele; Patouillard, Laure; Boulay, Anne-Marie; Bourgault, Guillaume; Bruille, Vincent de et al. (2019): IMPACT World+: a globally regionalized life cycle impact assessment method. In *The International Journal of Life Cycle Assessment* 24 (9), pp. 1653–1674. DOI: 10.1007/s11367-019-01583-0.

Calabrese, Edward J.; Baldwin, Linda A. (2003): Hormesis: The Dose-Response Revolution. In *Annual Review of Pharmacology and Toxicology* 43 (Volume 43, 2003), pp. 175–197. DOI: 10.1146/annurev.pharmtox.43.100901.140223.

Chae, Yooeun; An, Youn-Joo (2018): Current research trends on plastic pollution and ecological impacts on the soil ecosystem: A review. In *Environ Pollut* 240, pp. 387–395. DOI: 10.1016/j.envpol.2018.05.008.

Corella-Puertas, Elena; Hajjar, Carla; Lavoie, Jérôme; Boulay, Anne-Marie (2023): MarILCA characterization factors for microplastic impacts in life cycle assessment: Physical effects on biota from emissions to aquatic environments. In *J Cleaner Prod* 418, p. 138197. DOI: 10.1016/j.jclepro.2023.138197.

ECHA (2008): Guidance on information requirements and chemical safety assessment. Chapter R.10: Characterisation of dose [concentration]-response for environment. Edited by European Chemicals Agency. Helsinki (FI).

Fantke, Peter; Bijster, Marian; Guignard, Cécile; Hauschild, Michael; Huijbregts, Mark; Jolliet, Olivier et al. (2017): USEtox® 2.0 Documentation (Version 1.1).

Galafton, Christina; Thonemann, Nils; Vijver, Martina G. (2025): It is time to develop characterization factors for terrestrial plastic pollution impacts on ecosystems in life cycle impact assessment – a systematic review identifying knowledge gaps. In *Int J Life Cycle Assess* 30 (5), pp. 994–1010. DOI: 10.1007/s11367-025-02446-7.

Gigault, Julien; Halle, Alexandra ter; Baudrimont, Magalie; Pascal, Pierre-Yves; Gauffre, Fabienne; Phi, Thuy-Linh et al. (2018): Current opinion: What is a nanoplastic? In *Environ Pollut* 235, pp. 1030–1034. DOI: 10.1016/j.envpol.2018.01.024.

Gottschalk, Fadri; Kost, Elias; Nowack, Bernd (2013): Engineered nanomaterials in water and soils: A risk quantification based on probabilistic exposure and effect modeling. In *Environmental Toxicology and Chemistry* 32 (6), pp. 1278–1287. DOI: 10.1002/etc.2177.

Guo, Jing-Jie; Huang, Xian-Pei; Xiang, Lei; Wang, Yi-Ze; Li, Yan-Wen; Li, Hui et al. (2020): Source, migration and toxicology of microplastics in soil. In *Environ Int* 137, p. 105263. DOI: 10.1016/j.envint.2019.105263.

Horton, Alice A.; Walton, Alexander; Spurgeon, David J.; Lahive, Elma; Svendsen, Claus (2017): Microplastics in freshwater and terrestrial environments: Evaluating the current understanding to identify the knowledge gaps and future research priorities. In *Sci Total Environ* 586, pp. 127–141. DOI: 10.1016/j.scitotenv.2017.01.190.

Hurley, Rachel R.; Nizzetto, Luca (2018): Fate and occurrence of micro(nano)plastics in soils: Knowledge gaps and possible risks. In *COESH* 1, pp. 6–11. DOI: 10.1016/j.coesh.2017.10.006.

Lavoie, Jérôme; Boulay, Anne-Marie; Bulle, Cécile (2021): Aquatic micro- and nano-plastics in life cycle assessment: Development of an effect factor for the quantification of their physical impact on biota. In *J Ind Ecol*. DOI: 10.1111/jiec.13140.

Newman, Michael C. (2014): Fundamentals of Ecotoxicology: The Science of Pollution. 4th ed.: CRC Press.

OECD (2006): Current Approaches in the Statistical Analysis of Ecotoxicity Data: A guidance to application. Edited by OECD Publishing. Paris (OECD Series on Testing and Assessment, 54).

OECD (2016a): Test No. 220: Enchytraeid Reproduction Test. Edited by OECD Publishing. Paris (OECD Guidelines for the Testing of Chemicals, Section 2). Available online at https://doi.org/10.1787/9789264264472-en.

OECD (2016b): Test No. 222: Earthworm Reproduction Test (Eisenia fetida/Eisenia andrei). Edited by OECD Publishing. Paris (OECD Guidelines for the Testing of Chemicals, Section 2). Available online at https://doi.org/10.1787/9789264264496-en.

OECD (2016c): Test No. 232: Collembolan Reproduction Test in Soil. Edited by OECD Publishing. Paris (OECD Guidelines for the Testing of Chemicals, Section 2). Available online at https://doi.org/10.1787/9789264264601-en.

Owsianiak, Mikołaj; Hauschild, Michael Z.; Posthuma, Leo; Saouter, Erwan; Vijver, Martina G.; Backhaus, Thomas et al. (2023): Ecotoxicity characterization of chemicals: Global recommendations and implementation in USEtox. In *Chemosphere* 310, p. 136807. DOI: 10.1016/j.chemosphere.2022.136807.

Posthuma, Leo; Suter II, Glenn W.; Traas, Theo P. (2001): Species Sensitivity Distributions in Ecotoxicology https://doi.org/10.1201/9781420032314. 1st ed.: CRC Press.

Posthuma, Leo; van Gils, Jos; Zijp, Michiel C.; van de Meent, Dik; Zwart, Dick de (2019): Species sensitivity distributions for use in environmental protection, assessment, and management of aquatic ecosystems for 12 386 chemicals. In *Environmental Toxicology and Chemistry* 38 (4), pp. 905–917. DOI: 10.1002/etc.4373.

Redondo-Hasselerharm, Paula E.; Rico, Andreu; Huerta Lwanga, Esperanza; van Gestel, Cornelis A.M.; Koelmans, Albert A. (2024): Source-specific probabilistic risk assessment of microplastics in soils applying quality criteria and data alignment methods. In *J Hazard Mater* 467. DOI: 10.1016/j.jhazmat.2024.133732.

Rillig, Matthias C. (2012): Microplastic in Terrestrial Ecosystems and the Soil? In *Environ Sci Technol* 46 (12), pp. 6453–6454. DOI: 10.1021/es302011r.

Rillig, Matthias C.; Lehmann, Anika; Souza Machado, A. Abel de; Yang, Gaowen (2019): Microplastic effects on plants. In *New Phytol* 223 (3), pp. 1066–1070. DOI: 10.1111/nph.15794.

Rosenbaum, Ralph K.; Bachmann, Till M.; Gold, Lois Swirsky; Huijbregts, Mark A. J.; Jolliet, Olivier; Juraske, Ronnie et al. (2008): USEtox—the UNEP-SETAC toxicity model. Recommended characterisation factors for human toxicity and freshwater ecotoxicity in life cycle impact assessment. In *Int J Life Cycle Assess* 13 (7), pp. 532–546. DOI: 10.1007/s11367-008-0038-4.

Rozman, Ula; Kalčíková, Gabriela (2022): Seeking for a perfect (non-spherical) microplastic particle – The most comprehensive review on microplastic laboratory research. In *J Hazard Mater* 424, p. 127529. DOI: 10.1016/j.jhazmat.2021.127529.

Saadi, Nadim; Lavoie, Jérôme; Fantke, Peter; Redondo-Hasselerharm, Paula; Boulay, Anne-Marie (2025): Including impacts of microplastics in marine water and sediments in life cycle assessment. In *J Cleaner Prod* 520, p. 146037. DOI: 10.1016/j.jclepro.2025.146037.

Salieri, Beatrice; Stoudmann, Natasha; Hischier, Roland; Som, Claudia; Nowack, Bernd (2021): How Relevant Are Direct Emissions of Microplastics into Freshwater from an LCA Perspective? In *Sustainability* 13 (17), p. 9922. DOI: 10.3390/su13179922.

Souza Machado, Anderson Abel de; Lau, Chung W.; Kloas, Werner; Bergmann, Joana; Bachelier, Julien B.; Faltin, Erik et al. (2019): Microplastics Can Change Soil Properties and Affect Plant Performance. In *Environ Sci Technol* 53 (10), pp. 6044–6052. DOI: 10.1021/acs.est.9b01339.

Tunali, Merve; Adam, Véronique; Nowack, Bernd (2023): Probabilistic environmental risk assessment of microplastics in soils. In *Geoderma* 430, p. 116315. DOI: 10.1016/j.geoderma.2022.116315.

Tunali, Merve; Nowack, Bernd (2025): Towards including soil ecotoxicity of microplastics and tire wear particles into life cycle assessment. In *Ecotoxicol Environ Saf* 303, p. 118856. DOI: 10.1016/j.ecoenv.2025.118856.

UN Environment Programme (Ed.): Global Guidance for Life Cycle Impact Assessment Indicators and Methods (GLAM). Available online at https://www.lifecycleinitiative.org/activities/life-cycle-assessment-data-and-methods/global-guidance-for-life-cycle-impact-assessment-indicators-and-methods-glam/.

Vázquez-Vázquez, Brais; Lazzari, Massimo; Hospido, Almudena (2025): Terrestrial characterization factors for bio- and fossil-based plastics: microplastics ingestion and additives release. In *Waste Manag* 196, pp. 106–114. DOI: 10.1016/j.wasman.2025.02.008.

Vijver, Martina G. (2019): The choreography of chemicals in nature; beyond ecotoxicological limits. In *Chemosphere* 227, pp. 366–370. DOI: 10.1016/j.chemosphere.2019.04.068.

Wigger, Henning; Kawecki, Delphine; Nowack, Bernd; Adam, Véronique (2020): Systematic Consideration of Parameter Uncertainty and Variability in Probabilistic Species Sensitivity Distributions. In *Integrated Environmental Assessment and Management* 16 (2), pp. 211–222. DOI: 10.1002/ieam.4214.

Zantis, Laura J.; Borchi, Caterina; Vijver, Martina G.; Peijnenburg, Willie; Di Lonardo, Sara; Bosker, Thijs (2023): Nano- and microplastics commonly cause adverse impacts on plants at environmentally relevant levels: A systematic review. In *Sci Total Environ* 867, p. 161211. DOI: 10.1016/j.scitotenv.2022.161211.

Zhou, Yujie; Wang, Junxiao; Zou, Mengmeng; Jia, Zhenyi; Zhou, Shenglu; Li, Yan (2020): Microplastics in soils: A review of methods, occurrence, fate, transport, ecological and environmental risks. In *Sci Total Environ* 748, p. 141368. DOI: 10.1016/j.scitotenv.2020.141368.

Zimmermann, Lisa; Dierkes, Georg; Ternes, Thomas A.; Völker, Carolin; Wagner, Martin (2019): Benchmarking the in Vitro Toxicity and Chemical Composition of Plastic Consumer Products. In *Environmental Science & Technology* 53 (19), pp. 11467–11477. DOI: 10.1021/acs.est.9b02293.
